# Soluble Rank Ligand Produced by Myeloma Cells Causes Generalised Bone Loss in Multiple Myeloma

**DOI:** 10.1371/journal.pone.0041127

**Published:** 2012-08-29

**Authors:** Clive Henry Buckle, Evy De Leenheer, Michelle Anne Lawson, Kwee Yong, Neil Rabin, Mark Perry, Karen Vanderkerken, Peter Ian Croucher

**Affiliations:** 1 Department of Human Metabolism, University of Sheffield Faculty of Medicine, Dentistry and Health, Sheffield, United Kingdom; 2 Department of Haematology, University College London, London, United Kingdom; 3 Department of Anatomy, University of Bristol, Bristol, United Kingdom; 4 Department of Haematology and Immunology, Vrije Universiteit Brussel, Brussels, Belgium; 5 Garvan Institute for Medical Research, Sydney, Australia; Universidade Federal do Rio de Janeiro, Brazil

## Abstract

Patients with multiple myeloma commonly develop focal osteolytic bone disease, as well as generalised osteoporosis. The mechanisms underlying the development of osteoporosis in patients with myeloma are poorly understood. Although disruption of the RANKL/OPG pathway has been shown to underlie formation of focal osteolytic lesions, its role in the development of osteoporosis in myeloma remains unclear. Increased soluble RANKL in serum from patients with myeloma raises the possibility that this molecule plays a key role. The aim of the present study was to establish whether sRANKL produced by myeloma cells contributes directly to osteoporosis. C57BL/KaLwRij mice were injected with either 5T2MM or 5T33MM murine myeloma cells. 5T2MM-bearing mice developed osteolytic bone lesions (p<0.05) with increased osteoclast surface (p<0.01) and reduced trabecular bone volume (p<0.05). Bone volume was also reduced at sites where 5T2MM cells were not present (p<0.05). In 5T2MM-bearing mice soluble mRANKL was increased (p<0.05), whereas OPG was not altered. In contrast, 5T33MM-bearing mice had no changes in osteoclast surface or trabecular bone volume and did not develop osteolytic lesions. Soluble mRANKL was undetectable in serum from 5T33MM-bearing mice. In separate experiments, RPMI-8226 human myeloma cells were transduced with an human RANKL/eGFP construct, or eGFP alone. RPMI-8226/hRANKL/eGFP cells, but not RPMI-8226/eGFP cells, stimulated osteoclastic bone resorption (p<0.05) *in vitro*. Sub-cutaneous injection of NOD/SCID mice with RPMI-8226/hRANKL/eGFP or RPMI-8226/eGFP cells resulted in tumour development in all mice. RPMI-8226/hRANKL/eGFP-bearing mice exhibited increased serum soluble hRANKL (p<0.05) and a three-fold increase in osteoclast number (p<0.05) compared to RPMI-8226/eGFP-bearing mice. This was associated with reduced trabecular bone volume (27%, p<0.05), decreased trabecular number (29%, p<0.05) and increased trabecular thickness (8%, p<0.05). Our findings demonstrate that soluble RANKL produced by myeloma cells causes generalised bone loss, suggesting that targeting RANKL may prevent osteoporosis in patients with myeloma.

## Introduction

An important clinical feature of multiple myeloma is the development of a bone disease characterised by the presence of osteolytic lesions, bone pain and pathological fractures. Patients with myeloma also develop generalised bone loss, or osteoporosis, independent of the focal osteolytic bone lesions [1]. There is a two-fold increase in risk of osteoporotic fractures in patients with myeloma [2] and individuals with monoclonal gammopathy of underdetermined significance have an increased risk of axial fractures prior to development of myeloma [3]. Although it is widely recognised that increased osteoclastic resorption accounts for the development of osteolytic bone disease, the cellular and molecular mechanism responsible for the generalised bone loss is poorly understood. Furthermore, studies aimed at clarifying the importance of osteoporosis in the bone disease associated with multiple myeloma, as well as the mechanism(s) involved, will help provide the rationale for targeting this component of the disease.

The identification of the ligand for receptor activator of NFκB (RANKL) [4], [5], [6], and the demonstration that RANKL plays a critical role in normal osteoclast formation [7], raises the possibility that abnormal expression of this molecule may stimulate osteoclast formation and bone resorption in myeloma. RANKL expression is increased in bone marrow stromal cells in patients with myeloma [8], [9] and may also be expressed directly by both murine and human myeloma cells [10], [11], [12], [13], [14]. Furthermore, RANKL may be upregulated in T lymphocytes derived from the bone marrow of patients with myeloma [15]. The soluble decoy receptor for RANKL, osteoprotegerin (OPG), prevents RANKL-association with receptor activator of NFκB (RANK) and blocks osteoclast formation and bone resorption [10]. Studies have demonstrated that myeloma cells down-regulate OPG production in stromal cells and osteoblasts in a contact dependent manner [8], [9], [16] and that serum concentrations of OPG are decreased in patients with myeloma [17], . Furthermore, targeting RANKL can prevent the development of myeloma bone disease demonstrating that RANKL plays a pivotal role [9], [10], [20], [21].

RANKL exists principally as a membrane-bound molecule, although a soluble form (sRANKL) is generated by either proteolytic processing, or alternative mRNA splicing [22], [23], [24]. The soluble form of RANKL can induce osteoclastogenesis *in vitro* [5], [25] and may be produced by T cells isolated from patients with myeloma [15]. Furthermore, myeloma cells have been shown to express the mRNA encoding the sRANKL isoform [23] and increases in the concentration of sRANKL, and the ratio of sRANKL/OPG, have both been detected in the serum of patients with multiple myeloma. However, the importance of sRANKL and its role in the development of osteoporosis associated with myeloma bone disease remains unclear. Therefore, the aim of the present study was to determine whether sRANKL causes osteoporosis in murine models of multiple myeloma.

## Materials and Methods

### The 5T2MM and 5T33MM Models of Myeloma

The 5T2MM and 5T33MM murine models of myeloma originated spontaneously in C57BL/KaLwRij mice [26], [27]. 5T2MM or 5T33MM cells were isolated from bone marrow of disease-bearing animals, purified and injected into recipient mice [28]. These models closely reflect many aspects of the disease seen in humans. For example, they include the growth of myeloma in bone, the dependency of the myeloma on the bone microenvironment for its growth and survival, the development of a paraprotein that reflects myeloma burden and, in the case of 5T2MM, the characteristic osteolytic bone disease. Animals were housed under conventional conditions and had free access to food and tap water. All procedures involving these mice were approved by the local ethics committee at the Free University of Brussels (Belgium). Male C57BL/KaLwRij mice were injected with either 5T2MM or 5T33MM cells, or left un-injected (naïve). Serum paraprotein was monitored using standard electrophoretic techniques throughout the development of the disease [29]. All animals, including the respective uninjected, non-tumour bearing, control groups, were sacrificed at 12 weeks for the 5T2MM bearing mice (n = 12, naïve control n = 9) and 4 weeks for the 5T33MM bearing mice (n = 7, naïve control n = 9).

### NOD/SCID Xenograft Model of Myeloma

Following whole body γ-irradiation of NOD/SCID mice (3.0 Gy per mouse, at a rate of 1Gy per 21.0 secs, using an IBL 437C ^137^Cs gamma source, CIS BioInternational) at day −1, RPMI-8226 myeloma cells (American Type Culture Collection (ATCC), Manassas, VA, USA) were injected subcutaneously (10^7^ per mouse) at day 0 (mice aged 8 weeks at day 0, and all male). The following tumour cells were injected into irradiated mice: RPMI-8226/hRANKL/eGFP (n = 8/group), RPMI- 8226/eGFP (n = 8/group), RPMI-8226-wild-type (n = 7/group). Subcutaneous tumour progression was monitored via both eGFP-imaging (LightTools®, Synopsis Inc., Pasadena, CA) and caliper measurement and mice were sacrificed once the tumour had reached 1 cm^3^. All procedures involving these mice were undertaken at the University of Sheffield, with written UK Home Office licence approval (ref. PPL402901).

Radiogaphic, Densitometric and MicroCT Analysis of Myeloma Bone Disease To assess the number of osteolytic bone lesions induced by the presence of myelomacells the femora and tibiae were radiographed using a Faxitron x-ray system (Hewlett Packard, McMinnville, Oregon). The radiographs were scanned using a UMAX PowerLook 1100 scanner. Images were enlarged using Adobe Photoshop 5.0 LE software and the numbers of lytic bone lesions in the proximal tibiae and distal femora were counted manually on the digital image. Total bone mineral density (BMD) was measured by dual-energy x-ray absorptiometry (DXA) using a PIXImus scanner (Lunar, Madison, WI).

In some studies tibiae were scanned using a microCT scanner (Skyscan 1172) at 50 kV, 200 uA, using a detection pixel size of 4.3 um^2^ and a 0.5 mm aluminium filter, capturing images (×2) every 0.7° through 180° rotation. Reconstruction was performed using Skyscan Recon software and images analysed using Skyscan CT analysis software. A 1 mm^3^ volume of trabecular bone, 0.2 mm distal to the growth plate, was selected as a region of interest (ROI), and trabecular volume as a proportion of tissue volume (BV/TV, %), and trabecular number (Tb. N, mm^−1^) was assessed in this area.

### Histomorphometric Analysis of Myeloma Bone Disease

The femora, tibiae, calvariae and lumbar vertebrae were dissected free of soft tissues and processed for histological analysis. Bones were decalcified in EDTA and embedded in paraffin. Sections were cut and stained with haematoxylin and eosin. Trabecular bone area as a proportion of the total area (Cn.Ar/T.Ar,%) was determined in the distal femoral metaphysis and proximal tibial metaphysis and lumbar vertebrae, on a minimum of two separate sections, using a Leica QWin image analysis system (Leica Microscope Systems, Milton Keynes, UK). The medullary area in the calvariae was measured using the Leica Qwin system and expressed as a proportion of total tissue area or as an absolute area. To identify osteoclasts, sections were reacted for the presence of tartrate resistant acid phosphatase (TRAP) and counterstained with Mayer's Haematoxylin. The number of osteoclasts and/or the proportion of surface occupied by osteoclasts on the cortical-endosteal bone surface (N.Oc/CE; Oc.S/CE,%) were determined and, where appropriate, the trabecular bone surface (N.Oc/Ca; Oc.S/Ca,%).

### Measurement of Soluble RANKL and OPG in Serum

At sacrifice, blood samples were obtained from naïve animals (n = 8 as control for 5T2MM; n = 9 as control for 5T33MM) and animals bearing 5T2MM (n = 12) or 5T33MM (n = 7) tumour cells. Serum was separated by centrifugation and murine sRANKL, and murine OPG, were each measured by commercially available ELISA (R&D Systems, Abingdon, UK), according to the manufacturer's instructions.

### Production of RPMPI-8226/hRANKL/eGFP myeloma cells

cDNA encoding full-length human RANKL (hRANKL) was originally isolated from the SAOS2 osteosarcoma cell line (ATCC, Manassas, VA, USA). Its sequence corresponds to AF053712 (NCBI). hRANKL was cloned into the pCL10.1 bicistronic expression vector upstream of the sequence encoding enhanced green fluorescent protein (eGFP - *Aequorea victoria*), in which co-expression is controlled by the murine stem cell virus promoter. Self-inactivating lentiviral particles were produced as described previously [30]. Briefly, 293T human embryonal kidney cells (ATCC, Manassas, VA, USA) were co-transfected using a four plasmid-system, which introduced lentiviral *gag-pol* genes, the vesicular stomatitis virus envelope glycoprotein gene, reverse transcriptase and the expression cassette from pCL10.1. Titre was determined by limiting dilution on HeLa cells (ATCC, Manassas, VA, USA). RPMI-8226 human myeloma cells were plated at 5×10^4^ cells per well in six-well plates, then exposed to viral supernatant (MOI 10) for 12 hours, in the presence of polybrene (8 µg.ml^−1^). Transduced RPMI-8226 expressing hRANKL/eGFP, or eGFP alone, were expanded in culture, and used for *in vitro* and *in vivo* experiments.

### Detection of hRANKL expression

Transduction was assessed using flow cytometry (anti-hRANKL-PE, eBioscience Inc. Cat. No. 12-6619, mouse IgG2b-PE isotype control Cat. No. 12-4732), end-point polymerase chain reaction (PCR) (using primers directed at hRANKL: FOR 5′-TAGGAGAATTAAACAGGCCTTTC-3′, REV 5′-CAAAAACTGGGGCTCAATC-3′) and western blot (performed using anti-hRANKL, R&D Systems Cat. No. AF626, 0.1 ug.ml^−1^, and detected with anti-goat-HRP, Dako Cat. No. P0449, 1/30000; abcam anti-RNA polymerase II antibody, Cat. No. ab5408, 1/2000, detected with anti-mouse-HRP, Amersham Biosciences Cat. No. NA931V, 1/15000, was used as a loading control). ELISA, directed at hRANKL (Biomedica Gruppe, Wien, Austria) (performed according to the manufacturer's instructions), was used to quantify hRANKL in culture supernatant produced using transduced cells, as well as control cells. Where appropriate, SaOS2 and MG63 human osteoblast like cells (ATCC, Manassas, VA, USA) were included as bone cell controls.

### Determining whether over-expressed hRANKL is biologically active in vitro

Freshly isolated mouse peripheral blood mononuclear cells were plated in medium conditioned with RPMI-8226/hRANKL/eGFP cells, and supplemented with rmM-CSF at 25 ng.ml^−1^ (using 6-well plates). Media conditioned using either wild-type or eGFP- transduced cells were used as controls. Alpha-MEM, supplemented with rmM-CSF (see above) and rhRANKL at 0.5 ngml^−1^, in order to match the concentration detected in the conditioned medium, was included as a positive control. The number of TRAP-positive multinuclear (3+) cells was determined at 7, 14 and 21-day time points, via light microscopy.

In order to determine whether the multinuclear TRAP-positive cells could resorb bone, freshly-isolated mouse PBMC were cultured on dentine slices in medium conditioned with RPMI-8226/hRANKL/eGFP, as above (using 96-well plates). eGFP-only and wild-type controls were also included. At 21-days, slices were stained, using toluidine blue, and resorption pits visualised and scored, using light microscopy.

### Statistical Analysis

Data were expressed as the mean ± SEM. Comparison between groups was performed by Mann-Whitney U test, Chi-square analysis or Student's unpaired t test, as appropriate. Comparisons between distributions were performed using a Kolmogorov-Smirnov test.

## Results

### 5T2MM but not 5T33MM Myeloma Cells Promote the Development of Myeloma Bone Disease in the Tibiae and Femora in vivo

**Figure 1:**
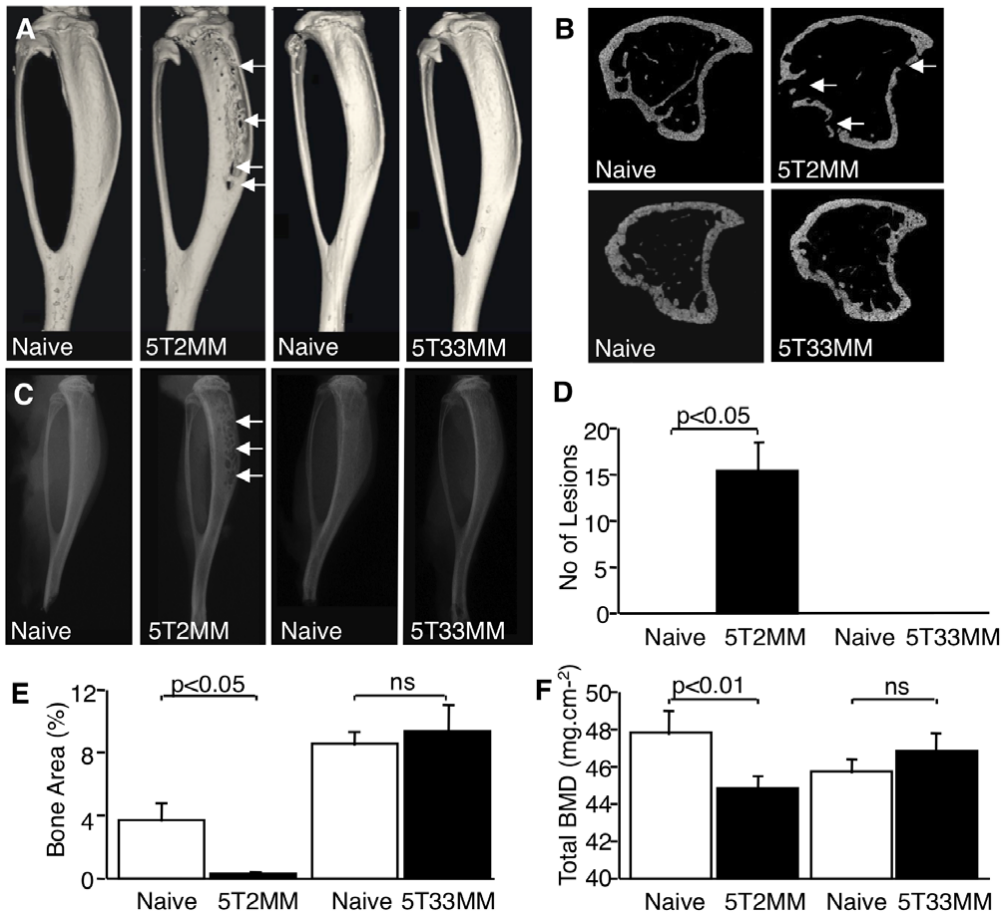
5T2MM, but not 5T33MM cells cause osteolytic bone disease and tumour-induced bone loss. A. Reconstructed 3-dimensional micro-CT images of the tibia of naïve mice, mice bearing 5T2MM cells and mice bearing 5T33MM cells. Lesions in the tibia of 5T2MM bearing animals are arrowed. B. Transverse sections of micro-CT images of tibiae from naïve mice, mice bearing 5T2MM cells and mice bearing 5T33MM cells. Lesions are arrowed. C. Radiographs of the tibia of naïve mice, mice bearing 5T2MM cells and mice bearing 5T33MM cells. Lesions are arrowed. D. Number of lesions in the tibia of naïve mice and 5T2MM or 5T33MM bearing mice. E. Trabecular bone area as a proportion of total tissue area in the tibia of naïve mice, and mice bearing 5T2MM or 5T33MM cells. F. Total bone mineral density of naïve mice, and 5T2MM or 5T33MM bearing mice. Statistical analysis by Mann-Whitney U test. Data = mean± S.E.M.

Injection of 5T2MM and 5T33MM cells into the tail vein of syngeneic C57BL/KaLwRij mice resulted in growth of myeloma cells in the bone marrow. Both 5T2MM and 5T33MM bearing mice had detectable levels of paraprotein in the serum (1.25±0.25 g.L^−1^, at 12 weeks, in the case of the 5T2MM-bearing mice). MicroCT, as well as radiographic analysis, of the long bones of mice bearing 5T2MM cells was used to demonstrate the presence of osteolytic bone lesions in the tibia and femur, which are areas in which increased osteoclast activity results in focal bone loss (Figure 1A–C). On a digitised radiographic image of a mouse tibia, the osteolytic bone lesions can be distinguished from surrounding intact bone as darker regions in which the bone has been removed. Assessment of the numbers of lesions in the tibia showed a significant increase in 5T2MM bearing animals when compared to naïve animals (p<0.05)(Figure 1D), whereas no lesions were observed in mice bearing 5T33MM cells (Figures 1C and 1D). Histomorphometric analysis of the long bones of 5T2MM bearing mice demonstrated a significant decrease in the trabecular bone area in the tibia and femur when compared to un-injected naïve animals (p<0.05). In contrast, trabecular bone area in mice bearing 5T33MM cells was not significantly different from naïve, uninjected mice (Figure 1E). Total BMD in the tibiae was also lower in 5T2MM bearing mice when compared to naïve, un-injected animals (p<0.01). There was no difference in total BMD in the tibiae in naïve mice and mice bearing 5T33MM cells (Figure 1F).

**Figure 2:**
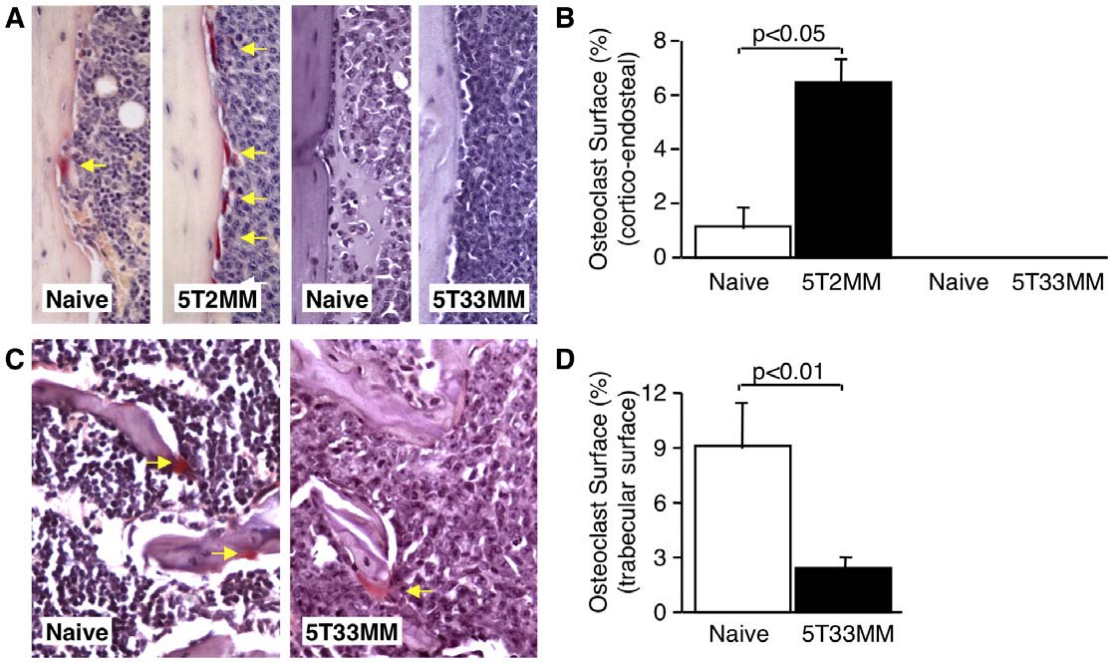
5T2MM, but not 5T33MM murine myeloma cells promote osteoclast formation in C57BL/KaLwRij mice. A. Photomicrographs of sections of the tibia reacted for TRAP activity from naive, 5T2MM and 5T33MM bearing mice showing the cortico-endosteal bone surface. TRAP-positive osteoclasts are arrowed. Original magnification ×40. B. The proportion of the cortical-endosteal bone surface occupied by osteoclasts, expressed as the percentage of the total bone surface, from naive mice and mice bearing 5T2MM or 5T33MM myeloma cells. C. Photomicrographs of sections of the tibia stained for TRAP activity from naive and 5T33MM bearing mice showing areas of trabecular bone. TRAP-positive osteoclasts are arrowed. Original magnification ×40. D. The proportion of trabecular bone covered by osteoclasts in naïve and 5T33MM bearing mice. Statistical analysis by Mann-Whitney U test. Data = mean± S.E.M.

Histomorphometric analysis demonstrated that the proportion of cortico-endosteal bone surface covered by osteoclasts was significantly increased in 5T2MM bearing animals when compared to control (p<0.05). In contrast, osteoclasts were not identified on the cortico-endosteal surface of naïve mice or mice bearing 5T33MM cells (Figure 2A and 2B). Analysis of the trabecular bone surfaces of 5T33MM bearing mice showed a decrease in the proportion of trabecular bone surface covered by osteoclasts (p<0.01) (Figure 2C and 2D). It was not possible to analyse the trabecular bone surface occupied by osteoclasts in 5T2MM bearing mice as the trabecular bone had been almost completely resorbed.

### Serum Concentrations of Soluble RANKL and OPG Are Abnormally Regulated in 5T2MM and 5T33MM Bearing Mice Compared to Non-Tumour Bearing, Naïve Mice

**Figure 3:**
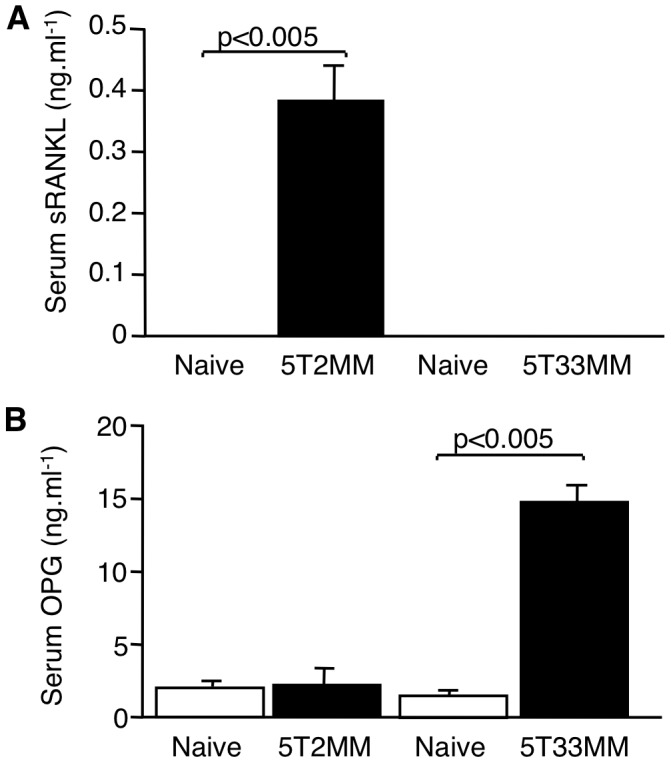
Serum concentrations of sRANKL and OPG are abnormally regulated in mice bearing 5T2MM and 5T33MM cells, respectively. Serum concentrations of sRANKL and OPG are abnormally regulated in mice bearing 5T2MM and 5T33MM cells, respectively. A. Serum concentrations of sRANKL in naive mice and mice bearing 5T2MM or 5T33MM cells. B. Serum concentrations of OPG in naive animals and animals bearing 5T2MM or 5T33MM cells. Statistical analysis by Mann-Whitney U test. Data = mean± S.E.M.

We next measured serum concentrations of sRANKL and OPG in mice bearing 5T2MM and 5T33MM cells and compared them to non-tumour bearing, naïve mice. sRANKL was not detectable in the serum of naïve mice or mice bearing 5T33MM cells. However, significant concentrations of sRANKL were measured in the serum of mice bearing 5T2MM cells (379.0±58.4 pg/ml, shown in Figure 3A). In contrast, serum concentrations of OPG in 5T2MM bearing animals and naïve animals were not significantly different (1.86±0.08 and 1.77±0.13 ng/ml respectively), whereas serum OPG was significantly increased in mice bearing 5T33MM cells when compared to naïve, un-injected animals (14.97±1.09 and 1.33±0.13 ng/ml respectively, p<0.005) (Figure 3B). There was no significant correlation between sRANKL and OPG in 5T2MM bearing mice.

### 5T2MM Bearing Mice Have Increased Bone Loss in the Vertebrae and Calvariae

**Figure 4:**
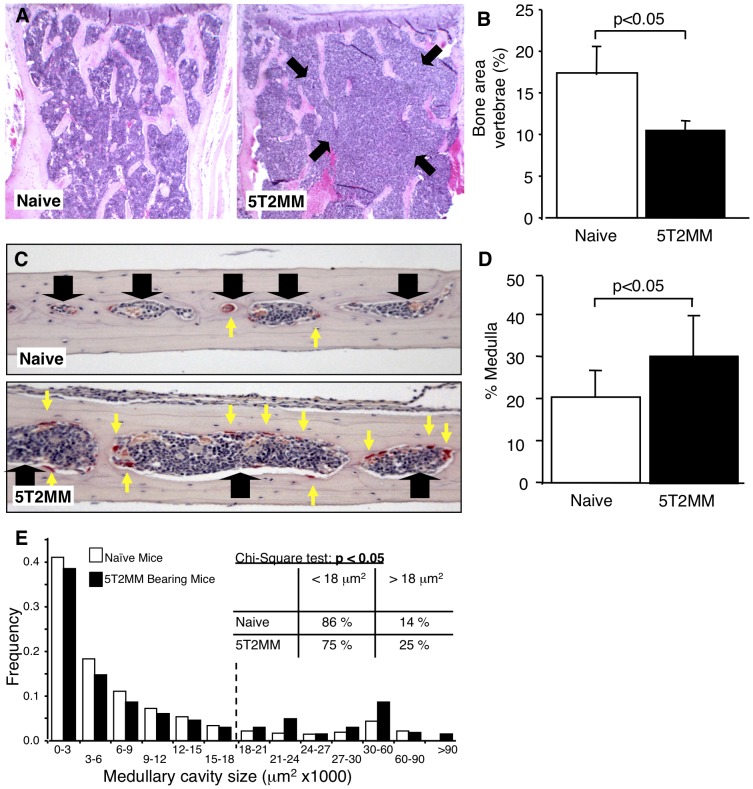
5T2MM bearing mice have increased bone loss in the lumbar vertebrae and calvariae. A. Photomicrographs of the vertebrae from naïve and 5T2MM bearing mice. Tumour infiltration is identified with black arrows. B. The proportion of trabecular bone area in the vertebrae from naïve and 5T2MM-bearing mice. C. Photomicrographs of the calvariae of naïve and 5T2MM bearing mice. Medullary spaces are identified with black arrows, and OC with yellow arrows. Non tumour-containing spaces are shown. D. The proportion of medullary area as a percentage of total tissue area in naïve and 5T2MM-bearing mice. E. Distribution of medullary spaces in naïve and 5T2MM-bearing mice, showing significant differences in distribution (KS test, p<0.02). Chi-square analysis demonstrated that 5T2MM-bearing mice had significantly fewer, smaller spaces and more, larger spaces than naïve mice (p<0.05). Data = mean± S.E.M. (B and D).

High serum concentration of sRANKL would be consistent with the bone loss observed in the tibia and the development of osteolytic bone lesions. However, alterations in circulating sRANKL would also be expected to promote more generalised bone loss. We therefore examined bones isolated from other sites for evidence of generalised osteoporosis in 5T2MM bearing mice. Histomorphometric examination of lumbar vertebrae demonstrated a significant decrease in trabecular bone in 5T2MM bearing mice compared to non-tumour bearing, naïve mice (Figure 4A and 4B), although, bone loss was also associated with significant infiltration of 5T2MM tumour cells. However, analysis of the medullary area in the calvariae demonstrated a significant increase in area in 5T2MM bearing mice compared to naïve mice (p<0.05), consistent with increased bone loss (Figure 4C, 4D). In the 5T2MM-bearing mice tumour infiltration was seen in only a limited number of medullary spaces (13%). Comparison of the medullary spaces from naïve and 5T2MM-bearing mice, but excluding those showing evidence of tumour infiltration, demonstrated a significant difference in the distribution of medullary space size (p<0.02) (Figure 4E). Chi-square analysis demonstrated that 5T2MM-bearing mice had significantly more, larger spaces and fewer, smaller spaces than naïve mice (p<0.05). In contrast, 5T33MM bearing mice showed no difference in the distribution of their medullary spaces when compared to naïve mice (p = 0.42; data not shown) and Chi-square analysis failed to show any difference in the proportion of larger medullary spaces when compared to naïve mice.

### RPMI-8226/hRANKL/eGFP human myeloma cells express biologically active soluble hRANKL

**Figure 5:**
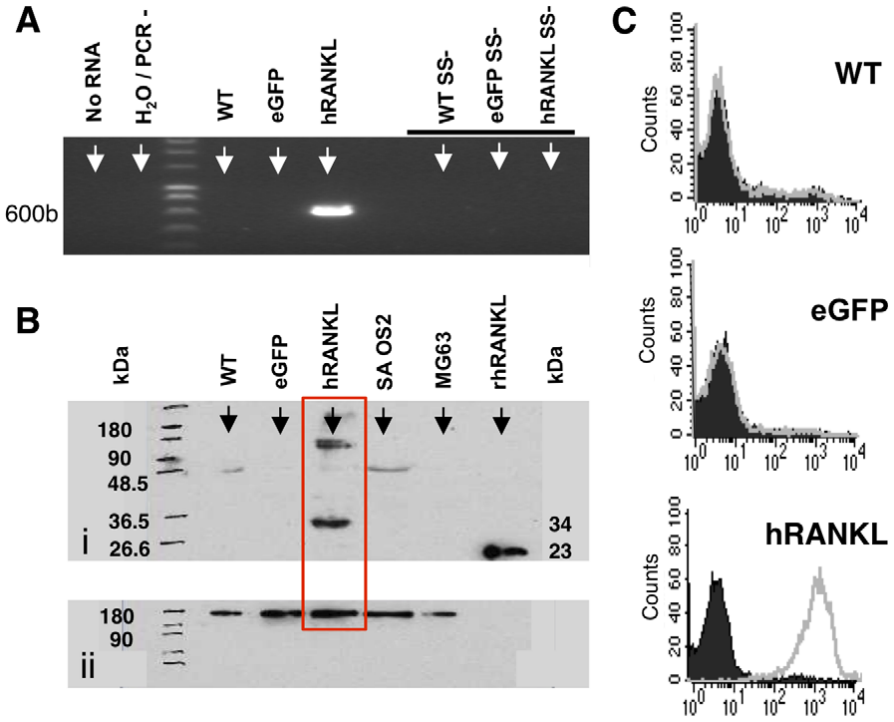
RPMI-8226/hRANKL/eGFP cells express hRANKL. A. End-point PCR demonstrating that human RANKL mRNA is present in RPMI-8226/hRANKL/eGFP cells (labeled hRANKL). hRANKL mRNA was not detected in the corresponding eGFP-only control cell line (labeled eGFP), or in wild-type cells (labeled WT). No product was amplified in the absence of RNA (labelled no RNA) or the superscript enzyme (labeled SS-). B. Western Blot analysis detected the hRANKL monomer (∼34 kDa) in culture supernatant from RPMI-8226/hRANKL/eGFP cells - red outline. (i) Wild-type and eGFP-only expressing cell controls are included, and the rhRANKL antibody-control is also shown (∼23 kDa). (ii) Ribosomal RNA loading controls were included. C. Flow cytometric analysis of the RPMI-8226/hRANKL/eGFP-expressing cells. Representative plots showing cells stained with anti-hRANKL-PE antibody (eBioScience, 1/50 dilution) (transparent profile) and isotype control (solid profile).

**Figure 6:**
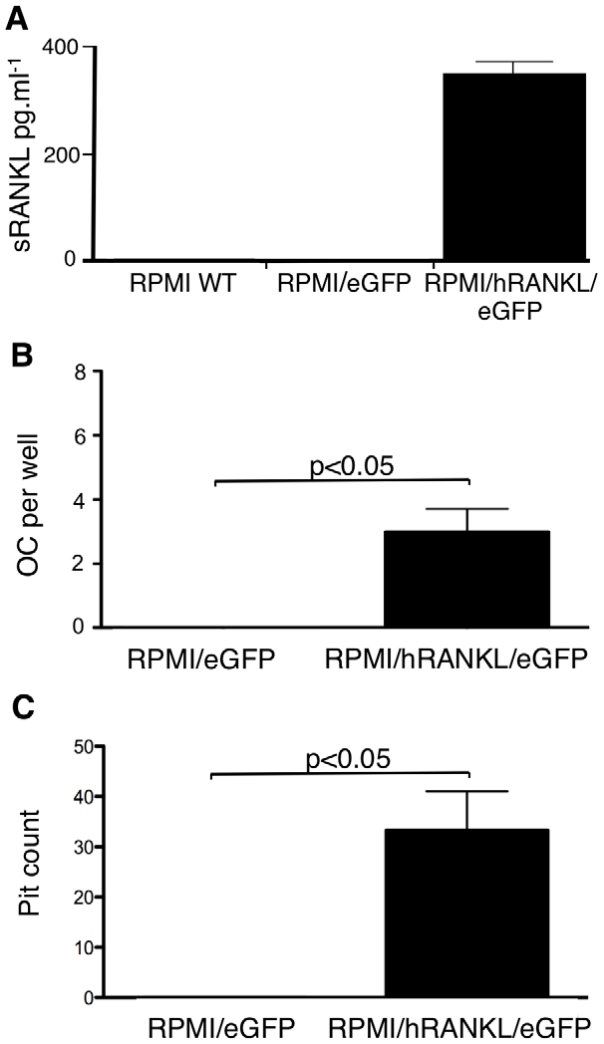
Transduced RPMI-8226 human myeloma cells express biologically active soluble hRANKL. A. shRANKL is detected by ELISA (Biomedica Gruppe, Wien, Austria), at a concentration of ∼0.4 ng.ml^−1^, in medium conditioned using RPMI-8226/hRANKL/eGFP cells, but not in controls. B. Multinucleated TRAP-positive cells (osteoclasts) formed in a culture of mouse PBMC cultured in medium conditioned with RPMI-8226/hRANKL/eGFP, supplemented with M-CSF (50 ng.ml^−1^). No osteoclasts were observed in control wells containing conditioned media from wild-type cells (data not shown) or RPMI-8226/eGFP. C. Resorption trails formed on the surface of dentine slices cultured in the presence of freshly prepared mouse PBMC, using medium conditioned with RPMI-8226/hRANKL/eGFP. No trails formed on slices cultured in medium conditioned by RPMI-8226/eGFP (or wild-type - data not shown). Statistical analysis performed using unpaired t-test. Data = mean± S.E.M.

To determine whether myeloma cell derived RANKL could induce systemic bone loss we generated myeloma cells over-expressing hRANKL (RPMI-8226/hRANKL/eGFP). Using end-point PCR, hRANKL mRNA was detected in RPMI-8226/hRANKL/eGFP (Figure 5A). In contrast, no expression was detected in the vector-only control, or wild-type cells. Following western blot, normalised using ribosomal RNA loading controls, hRANKL protein was detected in culture medium conditioned by RPMI-8226/hRANKL/eGFP cells (Figure 5B), but not in medium conditioned by wild-type or vector only control cells, as well as medium conditioned using osteoblast cell lines SAOS2 and MG63. In addition, flow cytometric analysis, using an antibody directed at hRANKL along with its corresponding isotype control, demonstrates that the RPMI-8226/hRANKL/eGFP cells express hRANKL at the cell surface (Figure 5C), whereas the RPMI-8226/eGFP and wild-type cells do not. Soluble hRANKL was detected in medium conditioned using the RPMI-8226/hRANKL/eGFP cells (circa 0.4 ng.ml^−1^), determined by ELISA. Soluble RANKL was not detected in wild-type or RPMI-8226/eGFP control cells (Figure 6A).

When freshly isolated mouse PBMC were cultured in medium conditioned with RPMI-8226/hRANKL/eGFP cells, multinucleate TRAP-positive cells were observed. No such cells formed in the cultures treated with medium conditioned using RPMI-8226/eGFP cells (Figure 6B). In separate *in vitro* experiments, where mouse PBMC were cultured on dentine slices, in the presence of medium conditioned by RPMI-8226/hRANKL/eGFP cells, resorption trails were observed (Figure 6C). No pits were observed on dentine slices cultured with medium conditioned using RPMI-8226/eGFP control cells (Figure 6C).

### RPMI-8226/hRANKL/eGFP-bearing mice have increased osteoclast number, elevated serum soluble RANKL and reduced trabecular bone volume and number

**Figure 7:**
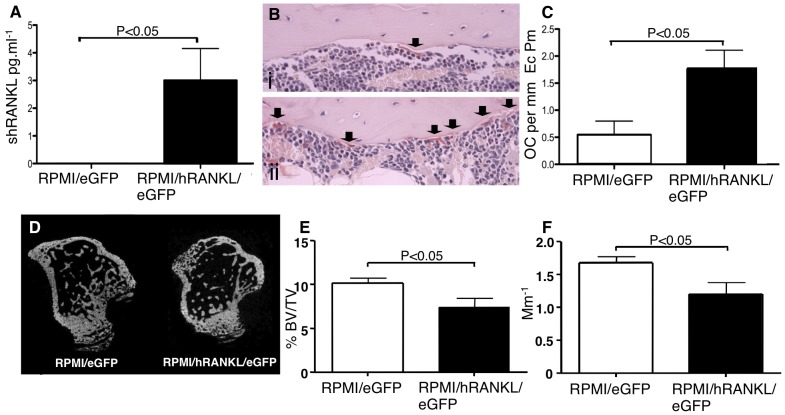
RPMI-8226/hRANKL/eGFP-bearing mice have elevated serum soluble RANKL, increased osteoclast number and reduced trabecular bone volume. A. ELISA demonstrating significantly increased serum sRANKL in mice bearing RPMI-8226/hRANKL/eGFP cells, compared to RPMI-8226/eGFP-bearing animals, as well as wild-type cells (data not shown). B. Photomicrographs of sections of mouse tibiae demonstrating that mice bearing RPMI-8226/hRANKL/eGFP (ii) have increased OC number at the CE-surface, compared to mice bearing RPMI-8226/eGFP control cells (i). OC are arrowed. Original magnification ×40. C. Increased OC number is observed at the CE surface in mice bearing RPMI-8226/hRANKL/eGFP cells. D. MicroCT images showing reduced bone volume in tibiae of mice bearing RPMI-8226/hRANKL/eGFP, as opposed to wild-type cells. E. RPMI-8226/hRANKL/eGFP-treated mice have decreased trabecular bone volume (27%) when compared to RPMI-8226/eGFP cell-bearing animals. F. RPMI-8226/hRANKL/eGFP-treated mice have reduced trabecular number (29%), when compared to RPMI-8226/eGFP-bearing animals. Statistical analysis performed using unpaired t-test. Data = mean± S.E.M.

Sub-cutaneous injection of NOD/SCID mice with RPMI-8226/hRANKL/eGFP, or RPMI-8226/eGFP cells, resulted in palpable tumour development in all mice. The mice bearing RPMI-8226/hRANKL/eGFP cells also exhibited increased serum soluble hRANKL (p<0.05) compared to RPMI-8226/eGFP cells (Figure 7A), although not all mice had detectable sRANKL. Following sacrifice, histological analysis of the tibiae from all mice showed that marrow space was not infiltrated with myeloma cells. However, histomorphometric analysis demonstrated that RPMI-8226/hRANKL/eGFP-bearing mice exhibited a three-fold increase in osteoclast number (p<0.05) along the cortico-endosteal surface compared to RPMI-8226/eGFP-bearing mice (Figure 7B and 7C). MicroCT analysis of tibiae of RPMI-8226/hRANKL/eGFP-bearing mice, with detectable sRANKL in the serum, was associated with reduced trabecular bone volume (27%, p<0.05) (Figure 7D and 7E), decreased trabecular number (29%, p<0.05) (Figure 7F), and increased trabecular thickness (8%, p<0.05 - data not shown).

## Discussion

In the present study we examined the ability of two syngeneic models of multiple myeloma, the 5T2MM and the 5T33MM models, to cause both osteolytic bone disease and osteoporosis. In 5T2MM bearing mice this was characterised by the presence of osteolytic bone lesions on radiographs, loss of trabecular bone, a reduction in total bone mineral density and an increase in the proportion of bone surface covered by osteoclasts. This mimics the features of the bone disease seen in patients with multiple myeloma. In contrast, injection of 5T33MM cells had little effect on bone. 5T33MM cells did not promote the development of osteolytic bone lesions, had no effect on trabecular bone area or total bone mineral density and did not increase osteoclast formation. Indeed, histomorphometric analysis demonstrated that osteoclast surface on trabecular bone was significantly reduced in mice bearing 5T33MM cells. These data contrast with a previous study in which 5T33MM cells were reported to promote the development of a myeloma bone disease [31]. The reason for the difference between the two studies is unclear. However, the 5T33MM cells have been maintained independently, in different laboratories for many years and therefore, this may reflect the selection of different clones of 5T33MM cells over time.

In addition to focal osteolytic bone lesions, generalised osteoporosis was observed in the bones of 5T2MM bearing mice in which tumour cells were not present. In the calvariae of these mice, significant bone loss was observed, which is consistent with the hypothesis that bone loss also occurs independently of local induction by tumour cells, and results from systemic induction of osteoclastogenesis. In support of this, studies have shown that a significant proportion of patients with multiple myeloma develop osteopenia or osteoporosis independent of lytic bone lesions [2]. The molecular mechanism responsible for the development of osteoporosis in myeloma is unknown.

Measurement of sRANKL in serum showed that 5T2MM-bearing mice had high circulating levels of sRANKL in all tumour-bearing mice. In contrast, sRANKL was not detectable in the serum of naïve mice, or mice bearing 5T33MM cells. This is consistent with the demonstration of increased serum concentrations of sRANKL in patients with myeloma and that an increase in the ratio of RANKL/OPG is associated with lytic bone lesions [32]. The source of sRANKL remains unclear, but may reflect production by cells within the bone marrow microenvironment, such as stromal cells and T-cells, or production by 5T2MM cells, since we have shown previously that these cells express RANKL [10]. Furthermore, the mechanism responsible for generating sRANKL is unknown, but may include proteolytic cleavage of a membrane-bound RANKL isoform, as myeloma cells can shed membrane bound molecules [33], [34] and express ADAM17, a proteinase capable of shedding RANKL [35]. Alternatively, circulating sRANKL may be generated by differential mRNA splicing, which has been reported in myeloma cells [13]. Treatment of the RPMI8226/eGFP/hRANKL cells with phorbol 12-myristate 13-acetate, which has been shown to activate membrane shedding activity, had no effect on membrane RANKL expression and did not increase sRANKL in the culture supernatent (data not shown). This may argue against proteolytic processing of the membrane bound form being the main mechanism of production. Irrespective of the source, these data support the notion that sRANKL, as a key regulator of osteoclastic bone resorption, drives the development of osteoporosis.

Serum concentrations of OPG were unchanged in 5T2MM-bearing mice, but were elevated in those mice bearing 5T33MM cells. This may account for the observed inhibition of osteoclast formation, the lack of detectable bone disease seen in the 5T33MM model and explain the difference between the present study and data reported by Garrett *et al.* [31]. The failure to affect BMD in 5T33MM bearing mice is likely to reflect the short timeframe of this model (4 weeks) and the relatively small contribution attributable to trabecular bone.

In order to investigate the hypothesis that osteoporosis is caused, at least in part, by elevated systemic sRANKL, experiments were performed using a mouse xenograft system in which the tumour cells were engineered to over-express hRANKL. Mice were injected subcutaneously with RPMI-8226/hRANKL/eGFP cells and growth shown to be restricted to the site of implant. Significant increases in serum sRANKL were observed in mice bearing RPMI-8226/hRANKL/eGFP cells, compared to control, myeloma cell bearing animals. Histological examination demonstrated that this increase was associated with elevated numbers of osteoclasts in bones from RPMI-8226/hRANKL/eGFP bearing animals, despite the absence of tumour cells in the bone marrow. These findings are associated with a significant reduction in bone volume and trabecular number in those animals. Taken together these data demonstrate that RANKL expressed by myeloma cells, and released as soluble RANKL, stimulates osteoclast formation and promotes bone loss at distant sites in the skeleton. In support of this, both OPG [10] and a soluble receptor activator of NF_k_B-Fc construct (data not shown) were able to prevent osteoclast formation and the development of bone disease in the 5T2MM model.

In conclusion, using mouse models of myeloma we have been able to demonstrate that myeloma is associated with both focal osteolytic bone disease and osteoporosis, and this is associated with increased serum concentrations of sRANKL. Furthermore, myeloma cells that express RANKL also produce soluble RANKL *in vivo* and this is able to promote osteoclast formation and osteoporosis. This supports the hypothesis that elevated serum soluble RANKL contributes to the development of osteoporosis in myeloma. Therefore, inhibiting RANKL may have therapeutic potential not only for the treatment of osteolytic bone lesions but also the generalised osteoporosis. Furthermore, this raises the possibility of using novel treatments such as the monoclonal antibody Denosumab, a fully human monoclonal antibody that targets the RANK-RANKL interaction [36]. This agent has been in development for the treatment of osteolytic bone disease in patients with multiple myeloma, for the treatment of bone disease in bone metastases and for osteoporosis in post- menopausal women [37], [38], [39]. Its role in the treatment of osteoporosis in myeloma is unclear but the data presented would argue that consideration should be given to its use in this setting. Thus, treatments such as Denosumab, which have been used successfully for the treatment of post-menopausal osteoporosis, could be considered as potential treatment of both the focal osteolytic disease [39], [40] and the generalised osteoporosis seen in patients with multiple myeloma.
